# Thermoresponsive and Fluorescent Polymers: From Nanothermometers to Smart Drug Delivery Systems for Theranostics Against Cancer

**DOI:** 10.3390/pharmaceutics17081062

**Published:** 2025-08-15

**Authors:** Mirian A. González-Ayón, Jesús E. Márquez-Castro, Diana V. Félix-Alcalá, Angel Licea-Claverie

**Affiliations:** Tecnológico Nacional de México, Instituto Tecnológico de Tijuana, Centro de Graduados e Investigación en Química, A.P. 1166, Tijuana 22414, BC, Mexico; mirian.gonzalez@tectijuana.edu.mx (M.A.G.-A.); jesus.marquezc201@tectijuana.edu.mx (J.E.M.-C.); m21210020@tectijuana.edu.mx (D.V.F.-A.)

**Keywords:** thermoresponsive polymers, fluorescence, nanogels, micelles, nanothermometers, drug delivery systems, bioimaging, sensors, theranostics

## Abstract

This mini-review article is focused on polymeric materials that comprise thermoresponsive and fluorescent organic units. The combination of fluorescent clusters/dots embedded in or grafted with polymers is not considered in this article. Here we review the preparation, characterization, and application of thermoresponsive polymers functionalized covalently with organic fluorescent compounds either compartmentalized or randomly distributed: block-copolymers, self-assembled micelles or vesicles, core–shell nanogels, and their temperature driven self-assembly/shrinkage/expansion and resulting effect in fluorescence: quenching, enhancing, shifting. The applications suggested for these smart-materials are reviewed in the last ten years and range from nanothermometers, drug delivery systems, agents for bioimaging, sensors, and advanced materials for theranostics focused on cancer treatment. This article is organized reviewing the preparation methods, the main characterization techniques, and the application, depending on polymer architecture and the emission wavelength of the fluorophores. Finally, comments, suggestions, and problems to be solved for the advancement of these materials in the future prior to real-life applications are given.

## 1. Introduction

The convergence of polymer chemistry, nanotechnology, and biomedical sciences has led to the development of multifunctional materials with extraordinary capabilities for disease diagnosis and therapy. Among these, thermoresponsive fluorescent polymers (TFP) represent a unique class of smart materials that respond to environmental stimuli, particularly temperature changes, while simultaneously offering real-time optical readouts. Their dual functionality makes them promising candidates for biomedical applications such as nanothermometers [1,2,3,4,5], drug delivery systems [6,7,8], agents for bioimaging [9,10,11], sensors [12,13], and advanced materials for theranostics [14,15], particularly in the context of oncology, where precise diagnostics and targeted treatment are critically needed.

The combination of thermal sensitivity and fluorescence opens new avenues for precision oncology. As nanothermometers, these polymers can provide real-time feedback during therapies such as photothermal or magnetic hyperthermia, allowing precise adjustment of the thermal dose to maximize tumor ablation. In addition to sensing temperature changes as nanothermometers, TFP have been used as sensors for other important biological compounds, such as ions. As contrast agents, these smart materials improve imaging accuracy by targeting specific tissues or cells and providing contrast without invasive procedures. Their responsiveness and tunable properties, both temperature response and fluorescence, make them valuable tools for biomedical applications.

The achievement of TFPs with precise control over polymer architecture, functionalization, and size has been accomplished through reversible addition–fragmentation chain transfer polymerization (RAFT) [4,5,10,16] or atom transfer radical polymerization (ATRP) [11,14]. However, more simple free radical polymerization methods (emulsion polymerization, surfactant free emulsion polymerization, dilution polymerization, and precipitation polymerization) has also allowed the production of TFP crosslinked systems with precisely tuned thermal transition thresholds and fluorescence properties [1,2,6,15,17]. These nanostructures, such as micelles [4,5,10,11,14], nano/microgels [3,7,8,9,12,13,15,17,18], and vesicles [16], exhibit optimized biodistribution, biocompatibility, and stimuli-responsive behavior, allowing them to be tested for in vivo applications.

**Figure 1:**
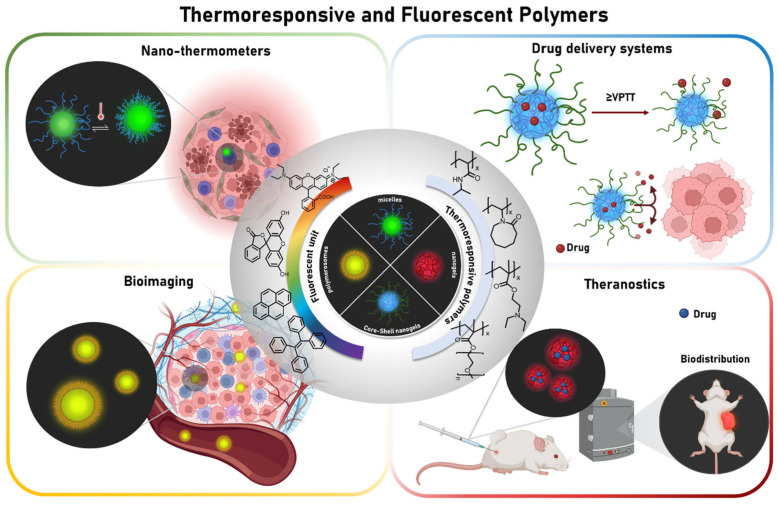
Thermoresponsive and fluorescent polymers and their main biomedical applications.

This mini-review article provides an overview of the state of the art, over the last ten years, on the production of TFPs for different biomedical applications. The physicochemical principles underlying thermoresponsiveness and fluorescence in polymers will be analyzed, along with strategies for integrating these functionalities into a single nanosystem and their biomedical applications, ranging from temperature mapping to theranostics (Figure 1). Special attention will be paid to design criteria, synthetic methodologies, and comments, suggestions, and problems to be solved for the future development of these materials prior to their practical application in cancer treatment.

## 2. Thermoresponsive Polymers

Polymers that undergo reversible changes in their main properties as response to variations in temperature are called thermoresponsive or thermosensitive polymers. These changes may involve alterations in solubility, phase, volume, conformation, or mechanical properties, around a specific temperature threshold known as the critical solution temperature. In the case that the polymer becomes insoluble above a critical temperature, the phenomenon is called lower critical solution temperature (LCST), while if the polymer precipitates below a critical temperature, the phenomenon is called upper critical solution temperature (UCST) [19]. Depending on the polymer architecture, these conformational changes in response to temperature can manifest in two ways: in LCST or UCST, manifesting as a reversible change in solubility passing from a completely soluble to an insoluble state (this is the case for linear or branched polymers); and as a volume phase transition temperature (VPTT) for covalently crosslinked polymer networks, where a reversible change in volume is observed, from a swollen to a contracted state. A common misconception in this field is to interchangeably refer to LCST or T_cp_ (Cloud Point Temperature). T_cp_ refers to the temperature at which a polymer solution at a specific concentration undergoes a phase transition from the soluble to the collapsed (aggregated) state, accompanied by clouding of the solution; while the LCST is the lowest T_cp_ value on the phase diagram. It is noteworthy that the cloud point curve in the phase diagram does not exactly coincide with the bimodal curve [20]. In both LCST and UCST cases, there is a balance between polymer–solvent and polymer–polymer interactions. The likelihood that a polymer chain will interact preferably with another polymer chain or with the solvent is determined by the temperature of the solution. Both hydrogen bonding and hydrophobic interactions in a polymer–water system are responsible for the phase transition. In a system polymer/water showing a LCST, there is a transition from hydrated random coil to a hydrophobic globule above this temperature; while in aqueous systems showing UCST, above this temperature the polymer interacts more with water through hydrophilic interactions.

**Figure 2:**
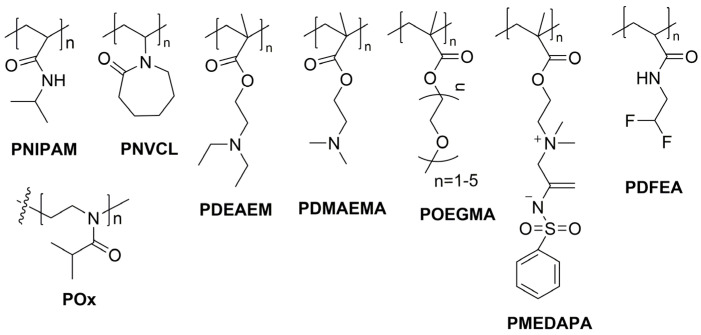
Main thermosensitive polymers functionalized covalently with organic compounds in the last ten years.

Thermoresponsive polymers have been studied over the years with a focus on various areas such as biomedicine, shape memory materials, separation sciences, and water management [21]. In particular, the area of biomedicine has been one of the most explored, specifically as drug delivery systems, in diagnostics, tissue engineering, and gene delivery [22]. In this sense, it is very important to tune the transition temperature for tailoring the thermal properties of thermosensitive polymers used in the biomedical applications mentioned. Copolymerization with monomers with different solubility in water has been used as a way to tune the transition temperature [23]. Furthermore, the transition temperature depends greatly on other factors such as concentration of the polymer, polymer molecular weight, and presence and concentration of salts, among others [24]. These reversible thermal transitions in polymers are not limited to aqueous solutions; however, only aqueous systems are of interest for biomedical applications. The widespread availability of polymers showing a LCST and the lack of stable UCST in aqueous systems makes LCST polymers more prevalent in biomedical applications [24]. In general, thermoresponsive polymers with LCST, could be classified as polyamides, polyalcohols, and polyethers, mainly [24]. The most studied and well-known thermoresponsive polymer is poly(*N*-isopropylacrylamide) (PNIPAM) [25], which belongs to the group of polyamides. PNIPAM has a LCST in water very close to the human physiological temperature, between 30 and 35 °C, which is very advantageous for biomedical applications. However, other LCST-type polymers, such as poly(ethylene glycol) derivatives, poly(*N*-vinylcaprolactam) (PNVCL) [26], poly(*N,N*-diethylaminoethyl methacrylate) (PDEAEM), and acrylamide derivatives, have also been tested in biomedical applications [21,27,28]. Various copolymers [29], blocks [30], hydrogels [31,32], core–shell nanogels [33,34], stars [35,36], microspheres [37], brushes [38], and nanocomposites [39] had been obtained based on thermoresponsive polymers. However, within the last ten years, thermoresponsive polymers covalently linked to fluorescent segments with nano/microgel and micelle-like structures and a single case of vesicle-like aggregates, have been reported. The thermoresponsive polymers used are shown in Figure 2. Besides PNIPAM and PNVCL, poly(2-isopropyl-2-oxazoline) (POx) [40], poly(*N,N*-dimethylaminoethyl methacrylate) (PDMAEMA) [7], poly(oligo(ethylene glycol) monomethyl ether methacrylate) (POEGMA) [14], and poly(2-((2-(methacryloyloxy)ethyl) dimethylammonio) acetyl) (phenylsulfonyl) amide (PDEMAPA) [15] have been reported.

## 3. Fluorescence in Polymers

### 3.1. Fluorescent Polymers

In the last ten years, fluorescent polymers have attracted tremendous research interest for applications, including chemical sensing, optoelectronic devices, and bioimaging, among others, due to their ease of signal detection and outstanding analysis capability [41,42,43,44,45]. Conventional fluorescent polymers can be easily constructed through different methods. Physical encapsulation is currently the most widely used method for preparing fluorescent polymer nanoparticles. These nanoparticles typically have a core–shell structure, in which the chromophore is housed in the core. The lack of chemical bonds between the polymers and the fluorescence-emitting group can lead to certain issues. In some cases, fluorescent material may leak from the polymer system or aggregate and become quenched. This factor can result in a reduced luminescent performance [42], which is why this article does not review this type of fluorescent systems. Instead, there are two methods to prepare polymer nanomaterials with covalently linked fluorophores: the first involves copolymerizing functional monomers and conventional monomers into chains, followed by covalent linking of fluorophores into some of the functional monomers. This preparation method requires a functional group matching between the copolymer nanoparticles and the fluorophores. Additionally, fluorescent materials can often experience fluorescence quenching when they are localized on the surface of the nanoparticles. The second approach involves copolymerizing fluorescent monomers with conventional monomers. One of the advantages of this latter method is that it results in a more uniform distribution of fluorophores within the fluorescent polymeric nanoparticles. However, an inconvenience exists: the size of fluorescent polymer nanoparticles is difficult to adjust because of the steric hindrance posed by the luminescent group attached to the monomer [43,44,45]. Fluorescent polymers are very important in biomedicine to visualize, detect, and analyze biological processes with high precision [46]. Fluorescence spectroscopy and microscopy techniques are powerful tools for studying these biological mechanisms, to study interactions between medical devices and tissues, and to monitor drug release [47]. In this sense, fluorescent markers permit monitoring polymers in cells and their biodistribution in vivo with spatial and temporal resolution. These fluorescent polymers may combine imaging and drug release properties, acquiring the potential ability to act as theranostic agents [42]. However, all these applications depend on the choice of fluorophores to label a specific polymer of interest [48,49]. There are some criteria to consider in the design of fluorescent nanoparticles, such as the physical properties of the luminescent group, which can drastically modify the properties of polymers, their hydrophobicity, size distribution, and surface charge. For instance, a fluorophore like cyanine may be incorporated to change the surface charge of the polymeric nanoparticles and thus the cell penetration capacity, their distribution in the biological system, as well as their retention and elimination results from the body [50]. Consequently, incorporating these fluorophores in thermoresponsive polymers can affect their water solubility and aqueous stability; and therefore, the temperature response can be shifted to lower temperatures [51].

### 3.2. Organic Fluorophores

**Figure 3:**
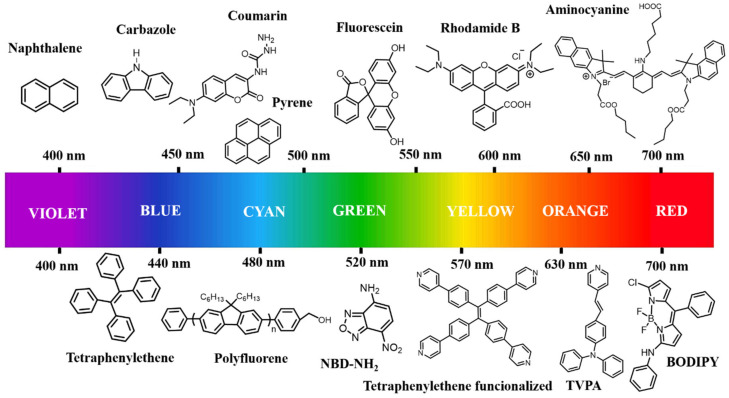
Light emission of conventional fluorophores from UV-visible to NIR infrared regions.

Fluorescence imaging is one of the most powerful bioanalytical techniques; it enables the visualization of biomolecules, it conformational changes, and its interactions inside living systems. A variety of advanced fluorescent imaging technologies and methods has been developed to support the growing requirements of scientific research in biology-related applications, e.g., positron emission tomography, computed tomography, single-molecule, multi-color, and second near-infrared (NIR-II) imaging. Molecular probes derived from common fluorophores (e.g., rhodamine B, fluorescein, naphthalimide, oxazine, xanthene, pyronin, rhodols, boron difluoride dipyrromethene (BODIPY), carbazole, and pyrene) have been used in these techniques [52,53,54]. These organic fluorophores exhibit high versatility in biomedical applications due to their properties, such as easy administration into cells, high sensitivity, and versatile design. Nevertheless, their practical application in imaging has been limited due to some drawbacks like, short absorption and emission wavelengths, low brightness, and poor photostability [55]. An additional problem is that most organic fluorophores exhibit low solubility in water; as a result, these fluorophores undergo aggregation-caused quenching (ACQ) in biomedical imaging processes. Furthermore, their photophysical properties such as emission, brightness, absorption, and quantum yields are of primary importance for in vivo applications and need to be determined. Various fluorophores can exhibit different excitation and emission spectra, ranging from UV-visible to near-infrared (NIR). Figure 3 shows commonly commercially available fluorophores; depending on substitution or modification, the emission band can be shifted within the spectrum. Meanwhile, fluorophores that emit in the visible region can be utilized rather than the ones emitting in the UV-region, as tissues obstruct excitation and emission of light in the UV-visible range due to absorption and scattering by mainly hemoglobin (Hb) and oxidized hemoglobin (HbO2), which might complicate acquisition of bioimages [56]. That is why, for in vivo applications, fluorescent nanomaterials emitting in the NIR range are more advantageous because tissues have two transparency windows, where the absorptions, auto-fluorescence, and light scattering are minimized. This property allows for real-time in-depth imaging (NIR-I 560–950 nm) [57] and (NIR-II 950–1350 nm) [58]. Finally, the cytotoxicity of fluorophores is often not considered since the relatively small amount of fluorophore required for polymer labeling leads to fluorescent polymers exhibiting low in vitro cytotoxicity.

### 3.3. Aggregation-Caused Quenching (ACQ) and Aggregation-Induced Emission (AIE)

Fluorescence quenching and signal switching are two principal changes that have been successfully utilized in nanoparticle bioimaging. Aggregation is a typical phenomenon that generally refers to the clustering of monomers at high concentrations of fluorophores [59]. In this context, fluorophores typically consist of planar and polycyclic π-conjugated structures, which form the basis for highly efficient luminescence. Generally, fluorophores are hydrophobic due to their aromatic nature. When the fluorophores are dispersed into aqueous media, they tend to aggregate since intermolecular interactions, such as π–π stacking, are increased, making it difficult for the molecules to disperse effectively. Aggregation typically results in a reduction or quenching of fluorescence. In summary, ACQ refers to the phenomenon where highly emissive fluorophores in dilute solution become less emissive or totally non-emissive in their aggregated state. ACQ is generally shown as unfavorable and poses a challenge for the application of conventional fluorophores [60]. However, all aromatic fluorophores exhibit the ACQ phenomenon, but the quenching efficiency may vary according to the molecular structure. For instance, the influence of the ACQ effect would not be noticeable if the aromatic rings were non-planar and could rotate freely. But aggregation does not always lead to ACQ; an opposite phenomenon of improving luminesce emission has been observed in concentrated solutions or solid states. This interesting phenomenon is known as aggregation-induced emission (AIE). Thang and co-workers discovered the new AIE effect in 2001. They studied a solution of 1-methyl-1,2,3,4,5-pentaphenylsilole in ethanol that significantly increased its fluorescence upon the addition of water. According to their investigations, this phenomenon was attributed to the formation of nanoparticle dispersions. Recently, AIE has gained significant attention because it is considered an advanced functional material for various practical applications, including bioimaging, stimuli-responsive sensing, and theranostics [61]. To develop a new AIE system, it is essential to understand the structural differences between AIE luminogens (AIEgens) and ACQ dyes. In contrast to the large planar structures of ACQ conventional dyes, most AIEgens exhibit highly twisted, propeller-like structures that are prone to intramolecular motion in the excited state. Intramolecular rotation in the excited state likely accelerates the non-radiative decay of the excitons to quench the emission of AIEgens in the solution state. Furthermore, since the intramolecular rotation is restricted in the aggregated or solid state, the non-radiative decay is reduced/stopped and an enhancement of luminescence is observed. The restriction of intramolecular rotation (RIR) is a proposed as a mechanism of AIE; however, other authors further propose the restriction of intramolecular vibration (RIV) as a supplement to RIR, since intramolecular vibration can also result in non-radiative decay. RIR and RIV are combined under the global term: restriction of intramolecular motion (RIM) to explain better the AIE mechanism. In solution active intramolecular motion, such as rotation and vibration, promotes non-radiative decay channels, to quench emission. While in an aggregate state, non-radiative decay channels are blocked due to the RIM, leading to enhanced emission. On the other hand, aggregated macromolecular ensembles (e.g., fluorescent block copolymers) can exhibit novel properties and functions that are not present in their molecular species. This discovery opened avenues for the development of new luminescent materials advancing the entire field of materials science [62,63].

## 4. Main Morphologies of Thermoresponsive and Fluorescent Polymers in the Nanometric Scale for Biomedical Applications

**Figure 4:**
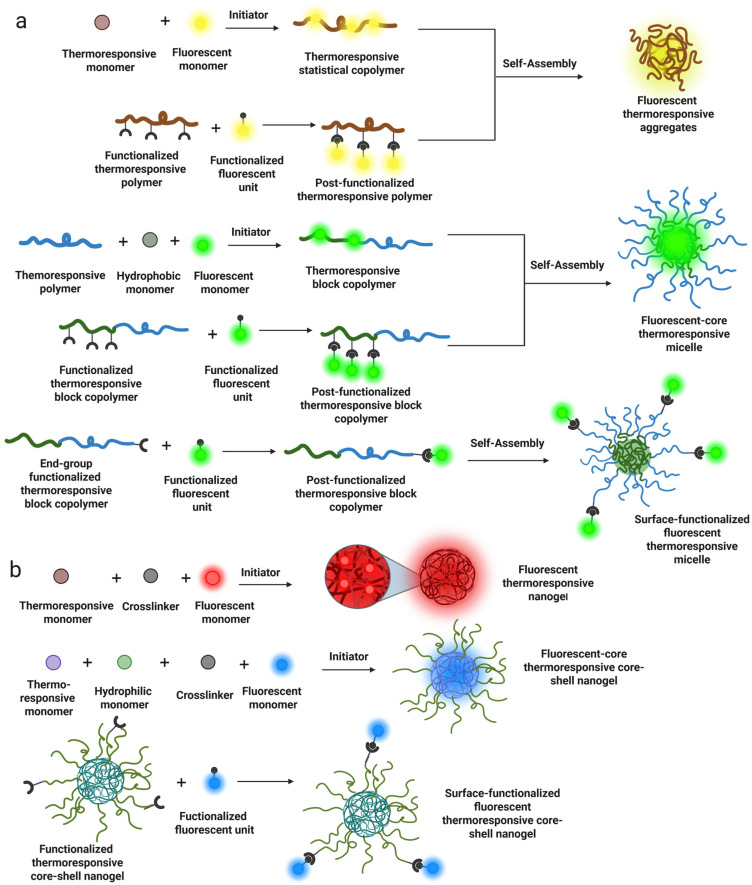
Strategies for preparation of different morphologies of thermoresponsive and fluorescent polymers in the nanometric scale: (**a**) self-assembling copolymers; (**b**) core–shell nanogels.

A general scheme highlighting preparation strategies of the main morphologies of thermoresponsive and fluorescent nanometric polymers can be seen in Figure 4.

### 4.1. Nanogels

Nanogels are three-dimensional network polymers chemically (covalent bonds) or physically crosslinked (hydrogen bonds, Van der Waals, and electrostatic interactions) of nanometric size. The properties of nanogels are mainly related to their capability to swell in different solvents and their permeable architecture [64]. Nanogels can be synthesized in one-step by free radical polymerization (FRP) using the methods of emulsion polymerization (EP) [7,13], surfactant free emulsion polymerization (SFEP) [6], dilution polymerization (DP) [17], and precipitation polymerization (PP) [8,65]. A two-step approach uses reversible addition–fragmentation chain transfer (RAFT) polymerization to obtain first thermoresponsive polymers [66,67] and subsequently post-polymerization/crosslinking of the first-prepared polymers by EP, SFEP, or PP methods [9]. Thermoresponsive nanogels respond to temperature changes in a medium and undergo a reversible phase transition (VPTT) of the network, shown as a swelling/deswelling process [19]. The encapsulation of fluorophores in nanogels has been widely reported [18,68,69], and although encapsulation is simple, it presents a disadvantage due to the possible release of them from the crosslinked matrix. This can be avoided with the fluorophore linked chemically to the network. Direct functionalization by in situ polymerization with a fluorescent comonomer or post-polymerization functionalization using a functional group (-SH, -NH_2_, -COOH) allows polymer–fluorophore covalent bonding to nanogels [44].

In recent years, thermoresponsive nanogels and their functionalization with fluorophores have been studied focused mainly on PNIPAM (see Table 1). The incorporation of fluorescent comonomers can shift the VPTT [2,7,8], and change the nanogel’s size with respect to the fluorophore content [2,65]. Control of size is relevant for biomedical applications; for instance, prolonged blood circulation and the enhanced permeation and retention effect (EPR) can be more successful for nanogels sizes around 100–200 nm [8].

The copolymerization of PNIPAM with other ionizable monomers achieves thermal and pH sensitivity, as PNIPAM-DMAEMA nanogels with adjusted VPTT of 40 °C, and the incorporation of acrylamido-fluorescein could allow tracking of cellular uptake [7]. Quantitative evaluation of fluorescence intensity as a function of temperature changes is relevant; Kim et al. reported the synthesis of microgels by copolymerization of NIPAM with comonomers of fluorescein and rhodamine B derivatives in the same chain as potential ratiometric sensors of temperature and pH. The emission of fluorescein and rhodamine B was affected at higher pH and lower pH, respectively, and fluorescence intensity shifted from green to orange emission [13].

Intracellular temperature can be sensed by cationic thermosensitive nanogels [2,3]; a temperature sensor using a fluorescent nanogel was prepared by Uchiyama et al. using a cationic free-radical initiator and benzothiadiazole as fluorophore [3]. The fluorescent intensity of the PNIPAM cationic nanogel (ζ=,+29 mZ) fluctuates in the physiological temperature interval (20–40 °C) and its performance was evaluated in HeLa cells [3].

AIE of fluorescence is reported when rigid and bulky naphtalimide molecules were introduced in PNIPAM nanogels with aggregation and restricted molecular motion; Zhang et al. obtained colloidal photonic crystals (CPCs) based on PNIPAM nanogels that show optical properties and disappeared above the VPTT of around 34–43 °C [12]. The fluorescence emission could be improved by adjusting the nanogel size, and the fluorescence intensity of CPCs at 450 nm was enhanced when the temperature increased from 25 to 49 °C; following nanogel contraction, these nanogels showed potential in fluorescence imaging applications [12]. Likewise, the incorporation of 5% of the fluorescent polymerizable naphtalimide derivative N-2-(6-(4-methylpiperazin-1-yl)-1,3-dioxo-1H-benzo[de]isoquinolin-2(3H)-yl-ethyl)acrylamide (FIM) in the synthesis of PNIPAM nanogels, achieves fluorescent covalently labeled nanogels with small sizes (10–20 nm); nanogels demonstrated potential in vitro tracking and internalization in murine neural stem cells with retention in cytoplasm for at least 24 h [17].

Another thermosensitive polymer similar to PNIPAM, of great interest for biomedical applications and exhibiting an LCST that can be adjusted to physiological values, is PNVCL. Banerjee et al. synthesized fluorescent and zwitterionic PNVCL microgels with core-corona morphology in a two-step method. In the first step rhodamine B-modified poly(glycidyl methacrylate) (RH-B-PGMA) was prepared via RAFT polymerization which acted as the macro-RAFT reagent during the synthesis of a block copolymer with poly(carboxybetaine) (PCB) (RH-B-PGMA-*b*-PCB). In the second step, this block copolymer was used as an emulsion stabilizer and macro-RAFT agent in an oil in water emulsion polymerization to prepare PNVCL based microgels with the addition of poly(ethyleneglycol diacrylate) (PEGDA, M_n_ = 250 g mol^−1^) as crosslinker. The fluorescent property and pH response of nanogels was achieved by the incorporation of RH-B units in PGMA polymer, accomplishing an incorporation of 35% and 90% of those units. PNVCL-based nanogels with different proportions of RH-B exhibit VPTT at 33 and 37 °C with reversible fluorescence intensity emission changes as a response at low pH (pH ≤ 5.5) [9].

Finally, the synthesis of zwitterionic thermoresponsive nanogels of poly(2-(2-(methacryloyloxy)ethyl) dimethylammonio)acetyl) phenylsulfonyl amide) (PMEDAPA), was reported by Peng et al. [15]. Nanogels incorporate the redox degradable crosslinker *N,N*-bis(acryloyl)cystamine (BAC), and formation of reactive -SH group allows a bonding site to Cyanine 5-maleimide. The emission of Cyanine 5 close to the NIR region allows visualization of in vitro and in vivo performance. Temperatures of response were determined at physiological intervals (32–43 °C), and nanogels showed an enhancement in penetration into cells and drug release properties by induced hyperthermia with microwave heating [15].

### 4.2. Self-Assembled Block Copolymers

#### 4.2.1. Thermoresponsive and Fluorescent Block Copolymers

**Figure 5:**
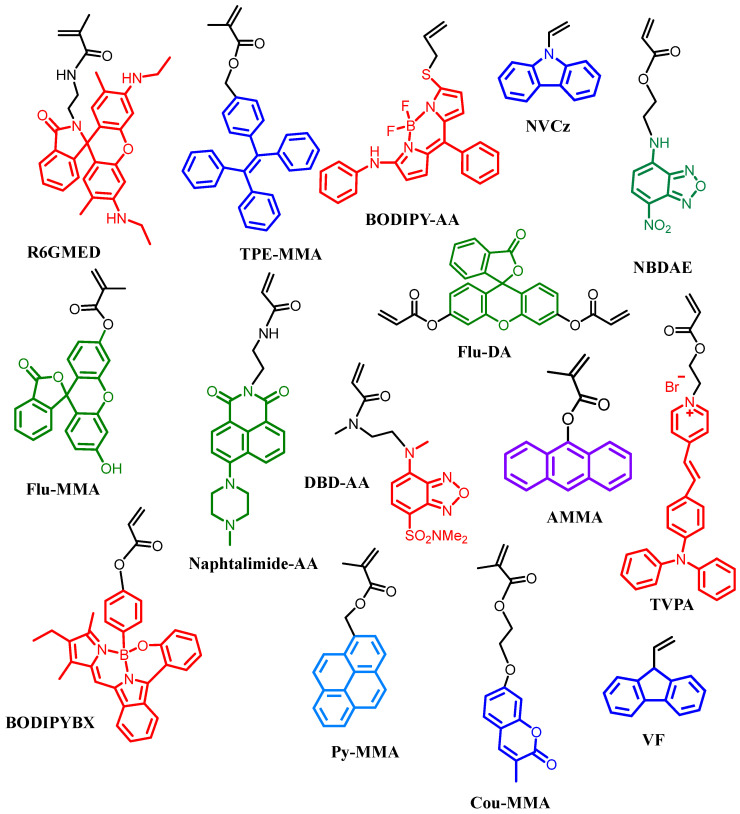
Examples of fluorescent monomers used for preparation of thermoresponsive and fluorescent copolymers in the nanometric scale; some of them are commercially available.

Block copolymers are a specific class of copolymers in which chemically distinct monomer units are grouped into discrete blocks along the polymer chain. For many decades, the self-assembly of small-molecule amphiphiles has been studied, revealing various morphologies, including spherical core–shell micelles, cylindrical micelles, and bicontinuous structures like lamellae and vesicles. Additionally, these self-assemblies can be activated by a thermoresponse [71]. The same type of self-assembled morphologies can be achieved using amphiphilic block copolymers. Thermoresponsive block copolymers are widely studied for the next reasons: First, temperature is closely related to various chemical reactions and physiological processes in nature; second, several methods are available for synthesizing thermoresponsive block copolymers, such as ATRP, RAFT, Group Transfer Polymerization (GTP), and Nitroxide-Mediated Radical Polymerization (NMRP) [72]. These polymerization methods allow for the obtention of thermoresponsive block copolymers with well-defined structures, precise molecular weights, and narrow molecular weight distributions. This accurate control in their structures facilitates the study of thermosensitive behavior in biological systems. Finally, the temperature sensitive characteristics are not easily disturbed by other stimuli. Consequently, monomers for thermoresponsive block preparation can be copolymerized with fluorescent monomers to form fluorescent self-assembled thermoresponsive nanoparticles. Figure 5 show examples of monomers containing fluorescent units that are used for the preparation of thermoresponsive and fluorescent copolymers/nanogels. The luminescent behavior of AIEgens depends on intramolecular motion, as discussed in another chapter. Therefore, the volume of thermoresponsive polymers in water changes with variations in the ambient temperature due to increased hydrophobic interactions.

As a result, AIE-active thermoresponsive copolymers can exhibit enhanced fluorescence behavior upon heating, because LCST showing thermoresponsive polymers become hydrophobic and undergo a volume collapse at high temperatures, restricting the intramolecular motions of AIEgens [73].

#### 4.2.2. Fluorescent Thermoresponsive Aggregates

Polymeric micelles are nanosized aggregates of block copolymers characterized by a core–shell structure, formed through the self-assembly of amphiphilic block copolymers in aqueous solution. In connection with this, the term “Critical Micellar Concentration” (CMC) refers to the minimum concentration of copolymers in solution required for micellar aggregate formation; by this definition, micelles remain stable at copolymer chain concentrations above the CMC but disassemble below this concentration [74]. According to existing reports on intracellular fluorescent temperature probes, various fluorescent temperature probes based on PNIPAM have been developed, showing morphologies such as micelles or vesicles. These probes incorporate AIEgens like polyfluorene (PF), rhodamine 6 derivative (R6GMED), triphenylethene (tPE), BODIPY, and 2-Acryloyloxyethylamino)-7-nitro-2,1,3-benzoxadiazole (NBDAE); in the fluorescent block. In these systems, the thermal response temperature can be adjusted between 29 and 36 °C; the difference in the thermoresponsiveness of PNIPAM and block copolymers containing PNIPAM arises from copolymerization with hydrophobic AIEgens. All the probes showed significant changes in fluorescence intensity when exposed to temperatures above 32 °C. Therefore, the thermoresponsive polymer defines the temperature range in which the fluorescent temperature probes can be effectively utilized for applications, such as bioimaging, drug release, sensors, and temperature sensing. In Table 2, different self-assembled fluorescent temperature responsive copolymers along with their applications can be found.

### 4.3. Other Thermoresponsive Copolymers and End-Functionalized Fluorescent Polymers

**Figure 6:**
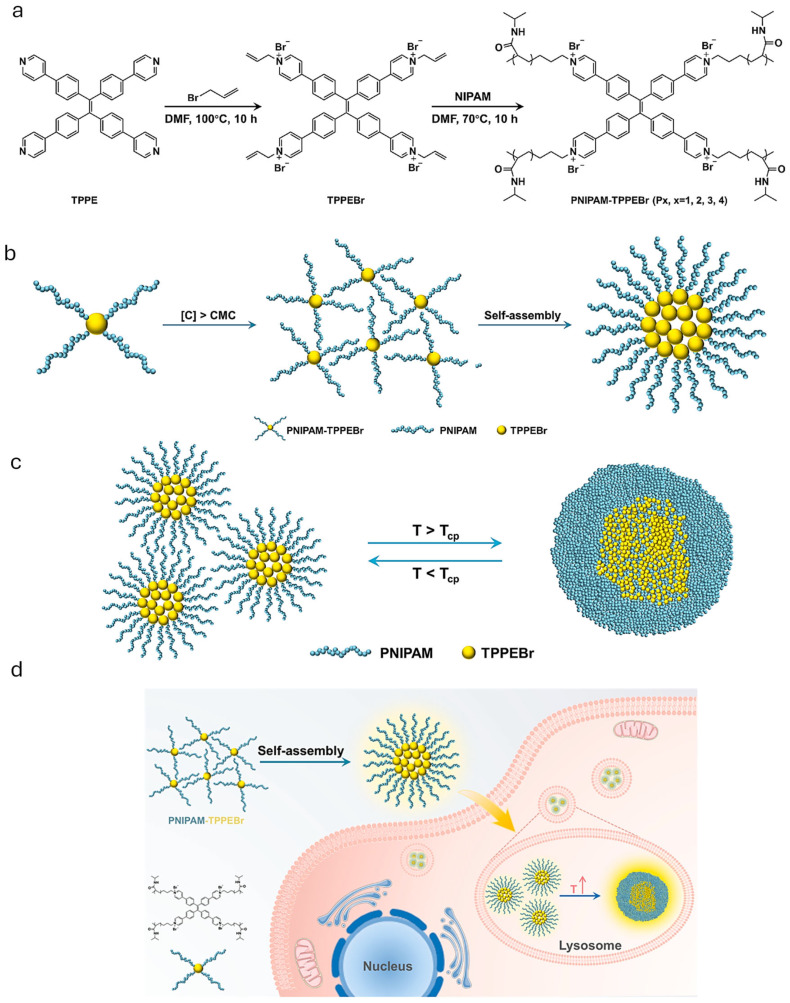
(**a**) Preparation route of TPPEBr and polymer PNIPAM_x_-TPPEBr (x = 1, 2, 3, 4). (**b**) Schematic illustration for self-assembly behavior of polymer PNIPAM-TPPEBr. (**c**) Schematic illustration for conformational change of PNIPAM-TPPEBr with increase of temperature. (**d**) Schematic representation of intracellular behavior of nanothermometer in A549 cells. Constructed using Figures published in [75], Copyright (2025), reproduced with permission from Elsevier.

Statistical copolymers with a majority of thermoresponsive units may self-assemble also to nanometric aggregates that may include fluorescent units as comonomers [48,76,79]; the aggregates formed are not as well defined as in the case of block-copolymers but depending on the desired application it may be sufficient. Another different approach is when a thermoresponsive polymer or copolymer is end-functionalized with fluorescent units [11,40,75,77,80]; in this case the self-assembly product may be well-defined [11,75] or not, depending on the specific case [Table 2]. One interesting example of this last methodology is the preparation of blue, green and red emitting fluorescent dyes based on the Cu(I)-catalyzed click reaction of azide end-functionalized poly(2-isopropyl-2-oxazoline) (POx) conjugated with three different acetylene fluorescent dyes: pyrene, BODIPY, and porphyrin, which are representative blue, green, and red fluorescence dyes [40]. Another one is the formation of a fluorescent-core-thermoresponsive (four arms) star-like polymer that assembles into micelles [75], as later discussed in Figure 6.

Another approach is the complexation of two polymers, one thermoresponsive and one fluorescent to nanosized aggregates [81]. In this example a cationic conjugated poly(fluorene-co-vinylene) (PFV) polymer forms the core, while temperature-responsive PNIPAM forms the thermoresponsive corona.

## 5. Biomedical Applications of Thermo- and Fluorescent Polymeric Nanoparticles

From the literature the applications envisioned for reported thermo-and fluorescent polymer nanomaterials are subdivided in the following subchapters: Nanothermometers as the original and main sensoric application, sensors comprising the sensing of analytes of biological significance, bioimaging (self-explanatory), drug delivery, where the use of the fluorophore is only to track the fate of the drug/drug carrier, and theranostics that comprise both bioimaging and drug delivery for diagnostic and therapy.

### 5.1. Nanothermometers

Temperature is one of the most crucial parameters for living organisms, significantly influencing various physiological processes and pathological activities, including cell division, gene expression, and anti-inflammatory response. Therefore, it is of great importance to achieve precise temperature sensing to understand these biological processes at the sub-cellular level. Among the different systems for temperature sensing, fluorescent nanothermometers rise significant attention due to their high sensitivity, non-invasive nature, excellent spatiotemporal resolution, and real-time responsiveness. Fluorescent nanothermometers are classified into two morphological types: fluorescent nanogel thermometers [1,2,3], which contain crosslinking units to form nano-scaled particles, and fluorescent linear polymeric thermometers with amphiphilic behavior [4,5,75]. For instance, Wang et al. developed a highly sensitive ratiometric fluorescent nanogel thermometer by radical polymerization. The nanogel consisted of a polarity-sensitive AIE dual-emission luminogen (TVPA) and a crosslinked thermoresponsive PNIPAM polymer, which enabled reversible phase transition. Additionally, the presence of a positively charged surface provided colloidal stability. In this case, when the external temperature was within the range of the LCST of PNIPAM, the AIE luminogen showed a reversible fluorescence switch between a blue-emitting and a red-emitting state, where a quantitative correlation with the external temperature for temperature sensing was established by using the emission intensity ratio as the variable parameter. Notably, the maximum relative thermal sensitivities of the optimized nanogel both in water and in physiological buffer were significantly higher than those of most organic fluorescent nanothermometers. The resultant nanogel was used to indicate the bactericidal temperature through both visual and ratiometric methods, demonstrating great promise for the rapid prediction of photothermal antibacterial effects [1].

On the other hand, Hayashi et al. reported cationic nanogels and cationic fluorescent nanogel thermometers based on PNIPAM [2]. These nanogels were synthesized by radical polymerization, incorporating a fluorescent DBD-AA unit. The nanogels exhibited size distributions from 230 to 314 nm at 25 °C, which decreased to 90–157 nm at 45 °C. The fluorescence intensity showed a notable increase associated with nanogel shrinkage and enhanced emission at around 32 °C, corresponding to the LCST of PNIPAM. The functionality of the cationic fluorescent nanogel thermometers in HeLa cells was similarly to the response in aqueous solutions; the nanogels introduced into HeLa cells showed clearly a fluorescence enhancement when the temperature of the culture medium was increased. In addition, the cationic fluorescent nanogel thermometer did not exhibit cytotoxicity since HeLa cells containing nanogels were able to undergo cell division similarly to unstained control cells [2].

Gong et al. in 2017, developed a nanothermometer using a simply structured copolymer hydrogel consisting of PNIPAM and a derivative of 4,4-difluoro-4-bora-3a,4a-diaza-s-indacene (BODIPY) units as thermoresponsive and polarity-sensitive fluorescent signaling parts, respectively [4]. The copolymer exhibited weak fluorescence in aqueous solutions at temperatures below 35 °C, whereas the red fluorescence intensity at 605 nm increased progressively with rising temperature, reaching a maximum at 47 °C. The biocompatibility of the nanothermometers was determined by a cellular cytotoxicity experiment, where different amounts of the copolymer were incubated with BHK cells for 24 h. Low cytotoxicity and good biocompatibility were obtained. The results showed that the fluorescence intensity in the red channel increased with the rising temperature [4].

Recently, Yin et al. reported a nanothermometer based on PNIPAM functionalized with the tetraphenylene fluorophore (TPPEBr) [75]. The phase transition behavior of PNIPAM in aqueous media from separated chains to dense core–shell micelle type spheres promoted the aggregation of TPPEBr fluorophores, resulting in enhanced fluorescence of the aggregates as the temperature increases. In addition, the phase transition of PNIPAM was associated with a decrease in the polarity of the microenvironment, causing this a blue shift in the emission wavelength of TPPEBr. The thermoresponsive performance of this nanothermometer was not affected by the intracellular microenvironment, and it was successfully applied in the temperature imaging of A549 cells. It was shown that when stimulated by ionomycin and an oxidative phosphorylation inhibitor, the cell temperature increased by approximately 1.5 °C and 1 °C, respectively. This luminescent sensor, provides a valuable method for developing a temperature sensing platform for biological systems [75] (Figure 6).

### 5.2. Drug Delivery Systems

Thermoresponsive polymeric nanocarriers improve the solubility and stability of hydrophobic drugs, protect them from interaction with the biological environment, and avoid their premature degradation, achieving prolonged blood circulation [15,83]. The drug release is triggered by temperature increment and additional characteristics, such as biodegradability at low pH conditions or by glutathione (GSH) presence in the medium, allowing a synergic temperature-degradation [8,15,84]. The target site could be reached by labeling the polymeric nanocarriers with ligands such as carbohydrates [82], amino acids [76,85], and transferrin [15]. Incorporating a fluorophore in the polymeric nanocarrier allows for the visualization and quantification of drug-loaded systems performance in vitro and in vivo.

One of the most studied anticancer drugs loaded into thermosensitive and fluorescent polymeric systems is Doxorubicin (DOX) [8,14,15,68,82]. DOX is widely used for the treatment of lung, breast, and bladder cancers; it shows a fluorescence emission (590 nm), which helps to visualize its release and internalization in cells by fluorescence microscopy. Fluorescent thermoresponsive PMEDAPA nanogels achieve 42.3% of DOX loading efficiency; in vitro drug release was improved at hyperthermic conditions (41 °C) with 49.3% of release after 48 h. This behavior was confirmed by cytotoxicity in HepG2 cells. Incubated empty nanogels show cytocompatibility even at 1000 µg mL^−1^, while DOX-loaded nanogels exhibit higher cytotoxicity at brief hyperthermia (41 °C) compared to normothermia (37 °C), related to the temperature-triggered DOX-release [15].

**Figure 7:**
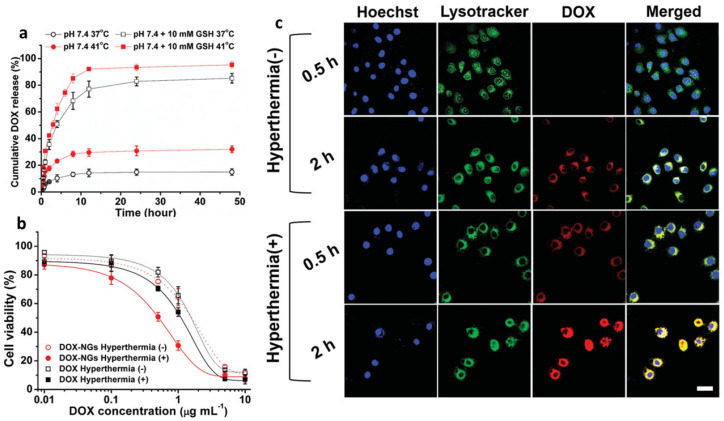
(**a**) DOX release from PNVCL-PHPMA NGs at different conditions; (**b**) cell viability of A5049 cells treated with DOX-loaded PNVCL-PHPMA NGs under normothermia or hyperthermia; (**c**) CLSM images of A5949 cells incubated with DOX-loaded PNVCL-PHPMA NGs under normothermia or hyperthermia for 0.5 and 2 h (scale bar 25 µm). Nucleus and lysosomes of A549 cells were stained with Hoechst (blue) and lysotracker Green (green), respectively. Constructed using Figures published in [8], Copyright (2015), reproduced with permission from Wiley.

On the other hand, Tian et al. designed biodegradable PNVCL nanogels with cleavable disulfide bonds using BAC as crosslinker [8]. These nanogels exhibit a hydrodynamic diameter (D_h_) around 89–196 nm and a good polydispersity index (PDI). VPTT was tailored from 35.8 to 44.3 °C by adding *N*-(2-hydroxypropyl)methacrylamide (HPMA) as a comonomer. Degradation of nanogels was verified adding from 10 to 40 mM of GSH in the medium through 12 h, and nanogels with VPTT of 40.2 °C were selected and loaded with DOX. Drug loading content was 12.7%, and release profiles were studied in normothermia (37 °C) and brief hyperthermia (41 °C) conditions with different concentrations of GSH. Release of DOX was improved up to 92% at conditions of hyperthermia and reductive environment with 10 × 10^−3^ mM of GSH through 12 h (Figure 7a). MTT cytotoxicity test confirmed that the half-maximal inhibitory concentration (IC_50_) was reached at a lower nanogel concentration (0.50 µg mL^−1^), inducing brief hyperthermia compared to the concentration required under normothermia conditions (1.58 µm mL^−1^) (Figure 7b). Fluorescein-labeled PNVCL-PHPMA nanogels (Flu-NGs) were incubated in A549 cells to confirm internalization and drug release; higher fluorescence intensity of DOX was observed in the cytoplasm after two hours of treatment under hyperthermia conditions (Figure 7c), and passive targeting was evaluated with intravenous injection of Cyanine 7.5 (Cy 7.5) labeled nanogels in mice with A549 tumor. A decrease in tumor growth was observed after treatment with nanogel injections after one hour and up to 24 h [8].

Fu et al. synthesized oligoethyleneglycol methacrylate (OEGMA) copolymers and galactose-derived monomer by RAFT polymerization, integrating Cy 7.5 with near-infrared fluorescence (795 nm) for targeted therapy against HepG2 cells [82]. Cy 7.5 labeling was done by functionalizing the terminal carboxyl group and reacting with a terminal amine in the block-copolymer. Self-assembled micelles were studied for DOX loading and release; In vitro release tests showed a 32% DOX release through 72 h, at pH 7.4 and 37 °C. Furthermore, the MTS assay results showed that galactose-labeled polymers had a higher affinity for HepG2 than unlabeled polymers, showing an IC_50_ of 113.7 μg mL^−1^ and 201.0 μg mL^−1^, respectively [82].

Deng et al. designed delivery systems based on triblock copolymers of PNIPAM, PF, and POEGMA (PF_11_-*b*-PNIPAM_120_-*b*-POEGMA_17_) prepared by ATRP, and these polymers assembled in core–shell–corona micelles that were prepared at 25 °C and 37 °C [14]. Temperature response was exhibited with an increase in the D_h_ of micelles from 30 to 47 nm at 33 °C. The DOX encapsulation and encapsulation efficiency (EE) of micelles were 3.5% and 38.5%, respectively. In vitro release tests were evaluated at 25, 37, and 40 °C; hyperthermia at 40 °C through 120 h allowed the release up to 85% of DOX, associated with destabilization and aggregation of micelles induced by temperature above the LCST. DOX-loaded micelles were incubated in HeLa cells at 25 °C, and cytotoxicity was determined by MTS assay; the IC_50_ of the micelles prepared at 25 °C and 37 °C was 63.47 µg mL^−1^ and 126 µg mL^−1^, respectively. The difference in cytotoxicity was associated with the stability of the micelles prepared at 25 °C and their higher DOX loading [14].

A study by Shakoory et al. reported a fluorescein-labeled PNIPAM-DMAEMA nanogel prepared with magnetic nanoparticles (MNPs) and loaded with cisplatin [7]. Loaded systems with a cisplatin-nanogel ratio of 1:10 exhibit a D_h_ increase from 90 to 282 nm and an EE of 29%; cisplatin loading occurs by coordination between the amine groups of DMAEMA and platinum atoms. The cisplatin release was improved by synergic pH-temperature effect at conditions of pH 5.8 at 40 °C; PNIPAM segment contracts at temperatures above its LCST and the pH-sensitivity of PDMAEMA is caused by the protonation of amine groups that would lose coordination ability. Results showed potential in hyperthermia-chemotherapy, but cytotoxic essays were not evaluated [7].

Encapsulation of bioactive compounds, like curcumin, has been reported for thermosensitive polymeric nanocarriers [17,84,85]. Santhamoorthy et al. synthesized a dual-stimuli-responsive copolymer of PNIPAM incorporating glycidyl methacrylate functionalized with L-lysine for targeted delivery [85]. Fluorescein isothiocyanate (FITC) was used to label the polymers; isothiocyanate reacts with the amine group in lysine units in the copolymer. The LCST of the copolymers was around 35 °C, and the formation of micro-size auto-aggregates was shown at high temperatures (45 °C). The curcumin loading in copolymer systems was ~69%, and the EE was ~70%, attributed to amine and carboxylic acid groups present in the copolymer interacting with curcumin. Drug release behaviors were evaluated under different pH and temperature conditions, and a synergic effect was shown in the evaluated systems. The drug release percentage was superior at the highest temperature (45 °C) and lower pH (~4), reaching approximately 80% in 12 h. The loaded systems were incubated in HepG2 and showed performance similar to free curcumin and concentration-dependent cytotoxicity determined by the MTT test [85].

Papadimitriou et al. synthesized fluorescent PNIPAM-based nanogels and micelles with a naphthalimide derivative covalently linked as nanocarriers of retinoic acid and for tracking in neural stem cells [17]. Nanogels loaded with 4.75% retinoic acid had an EE of 35.9%; meanwhile, micelles resulted in lower retinoic acid encapsulation and EE, with 0.11% and 92%, respectively. The PNIPAM-based nanogels were selected for subsequent experiments due to their stability and thermoresponsive behavior. Thermoresponsive studies confirm that nanogels exhibit a response around 37 °C in water and 38 °C in DMEM culture medium. The pharmacological effect of retinoic acid nanogels was evaluated on endogenous neural stem cells, at a concentration of 70 μg ml^−1^, and incubated for 48 h at 37 °C. Authors demonstrate differences between free retinoic acid and loaded nanogels; retinoic acid nanogels showed easy dissolution in aqueous media and also do not affect the viability and self-renewal of neurons [17].

### 5.3. Bioimaging

Medical imaging methods are very important for the detection and monitoring of many diseases, including cancer. However, its application is limited by low sensitivity, lack of specificity and targetability of current bioimaging probes [41,86]. Imaging of probes in single cells, tissues, and organs in vivo, requires a good contrast agent that is targeted to specific intracellular or extracellular molecules and that generates signals detectable by bioimaging devices. In this regard, various organic and inorganic materials have been widely studied as fluorescent agents, such as fluorescent dyes, quantum dots, and proteins. However, their low water solubility, high toxicity, and rapid photobleaching limits them for in vivo applications. In order to take advantage of the fluorescent properties and small size of the contrast agents mentioned above, more recent research has focused on coating these materials with biocompatible polymeric structures, such as dendrimers, block copolymers and branched/graft polymers [86]. Nevertheless, the focus of this work is specifically on thermoresponsive polymers covalently functionalized with organic fluorescent compounds, either compartmentalized or randomly distributed in nanogels and block copolymers self-assembled into micelles or vesicles. In this regard, there are only few reports on thermosensitive and fluorescent polymers for bioimaging applications.

Yamada et al. in 2015 reported the development of environmentally responsive fluorescent polymers via free radical polymerization of NIPAM with a fluorescent monomer based on either fluorescein (Flu), coumarin (Cou), rhodamine (RH), or dansyl (DA) in their skeleton. The polymers displayed a LCST at around 30 °C, and a fluorescence strength changing from blue to green close to this value. Uptake studies were performed in RAW264.7 cells. The PNIPAM LCST showed a crucial role in cellular uptake. No uptake was observed below the LCST but uptake increased above it. Cellular uptake of PNIPAM-Cou and PNIPAM-RH conjugated to L-a-phosphatidylethanolamine, dioleoyl (DOPE), a fusogenic lipid, was also increased. These lipid-conjugated fluorescence probes are expected to be useful as physiological indicators for intracellular imaging [79].

Wang et al. in 2025, reported the synthesis of TPE-NIPAM temperature-sensitive organic nanoparticles with AIE effect, by ATRP [11]. The nanoparticles can tune their fluorescence by changing temperature, emitting strong in the blue-green range. They are rapidly internalized by HeLa Cells and show no cytotoxicity.

In 2018, Koluchova et al. reported the synthesis of core–shell thermoresponsive and fluorescent nanogels through the self-assembly of amphiphilic copolymers obtained by two successive RAFT polymerizations [70]. The copolymers contain a hydrophilic biocompatible block of poly[*N*-(2-hydroxypropyl)-methacrylamide] (PHPMA) or poly(2-methyl-2-oxazoline) (PMeOx) and a thermoresponsive fluorinated poly[*N*-(2,2-difluoroethyl)acrylamide] (PDFEA). The in vitro ^19^F Magnetic Resonance Image experiments reveal the excellent sensitivity of the copolymer contrast agents. PHPMA-PDFEA (HF3) copolymer shows a stronger temperature dependence showing an increase in the magnetic resonance signal with increasing temperature, while for PMeOx-PDFEA copolymer the temperature dependence was not so significant at 30 °C and higher temperatures. In general, for both copolymers the signal intensity did not change significantly at 37 and 45 °C. The nanogels were suggested to be promising for angiogenesis imaging [70].

**Figure 8:**
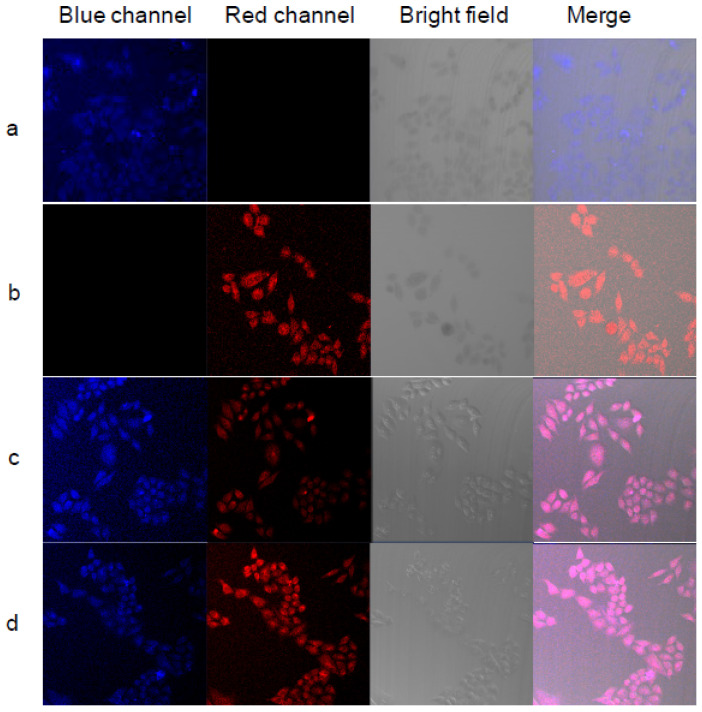
Fluorescent images of HeLa cells incubated with different polymers: (**a**) PNB; (**b**) PNmR; and (**c**,**d**) PMIX; (**a**–**c**) were observed at 25 °C and (**d**) was observed at 37 °C. (Reprinted with permission from authors in reference [48]).

PNIPAM and poly(*N*-isopropylmethacrylamide) (PNIPMA) were synthesized with different temperature responses (between 25 and 50 °C). A blue fluorescence molecule (7-[4-(trifluoromethyl)coumarin]methacrylamide) (TCMA) for PNIPAM-based polymers, and a red fluorescent molecule, BOBPYBX, was used with PNIPMA-based polymers [48]. Fluorescent images of HeLa cells incubated with the polymers PNIPAM-TCMA (PNB) and PNIPMA-BOBPBYX (PNmR) were explored (Figure 8). Figure 8a shows blue emission from the cells due to the uptake of PNmR. Figure 8b shows red emission from the cells due to the uptake of PNmR. Figure 8c,d show blue and red emissions, and the merged image show a pink emission. In Figure 8c, a stronger blue emission and a weaker red emission can be observed compared to Figure 8d, due to the change in observation temperature from 25 to 37 °C. The change in fluorescence as a consequence of LCST makes these materials attractive for applications, such as fluorescent thermometers and bioimaging of biological processes in organelles [48].

NIPAM-based block copolymers with 2-(diisopropylamino)ethyl methacrylate (DPA) and tetraphenylethylene (TPE) were obtained via RAFT polymerization as materials with potential applications in bioimaging. The polymers self-assemble reversibly into micelles showing AIE of fluorescence, which could be activated either by pH or temperature stimulus. The materials showed a response temperature at around 36 °C and blue emission. As the fluorescence intensity of the systems gradually increased with increasing solution pH or solution temperature, all this is in a reversible manner [10].

Microgels based on PNVCL were obtained using poly(carboxybetaine) (PCB) as a RAFT macro-agent and a rhodamine derivative as a fluorophore via emulsion polymerization. The microgels displayed an LCST at 33 °C, a UCST at 12 °C, and fluorescence emission at 580 nm (reddish-orange color) [9]. It is important to note that the aforementioned reports, which focused on the application of thermosensitive and fluorescent polymers in bioimaging, only address their interaction with the cell membrane (target) and their cellular internalization. Only two in vivo studies have been found for this type of polymer system in the last ten years and they will be addressed in the theranostics section.

### 5.4. Sensors

A straightforward application of thermoresponsive polymers as sensors is their usage as temperature sensors “thermometers”; in this review, Section 5.1 is devoted to describing their use as nanothermometers. In addition, thermoresponsive and fluorescent polymers have been used for sensing of other biologic important compounds, like ions. For instance, Kim et al. reported a microgel of poly(NIPAM-*co*-FLU-*co*-RH) crosslinked with *N,N*-methylenebisacrylamide (PNFR microgel) as a pH-sensor [13]. The PNFR microgel was able to monitor a wide range of pH, in which the FLU and RH units in the microgel showed independent emission changes within the range of pH studied. On the one side, FLU shows a strong green fluorescence with opening of its lactone ring under basic or neutral pH conditions, but the emission is strongly reduced at acidic pH, due to ring closure. On the other side, RH gives a strong orange fluorescence triggered by the opening of its lactam ring at acidic pH, while it becomes non-fluorescent when the ring is closed at basic or neutral pH. The changes in turbidity at temperatures above the transition temperature of the microgel provides a possible monitoring of the temperature as well.

Kong et al. have reported the use of PNIPAM containing 1,8-naphthalimide and rhodamine 6G moieties as chemosensors by photoinduced electron transfer (PET). Rhodamine 6G is the acceptor of energy transfer and the 1,8-naphthalimide moiety with piperazine group is the donor of energy transfer in the, by RAFT prepared copolymer [76]. The authors report photoluminescence (PL) peaks at 520 nm and 555 nm originated from the 1,8-naphthalimide and rhodamine 6G moieties in the copolymer, respectively. The 520 nm PL intensity (I_520_) is enhanced with increasing H^+^ concentration (pH < 3.5) in Britton–Robinson (B-R) buffers. The 555 nm PL intensity (I_555_) is dependent on the pH value of the B-R buffer, the concentration of Fe^3+^ ions and temperature for excitation at 400 nm. The ratio of the emission from the acceptor to that of the donor (I_555_/I_520_) is sensitive to the Förster-type resonance energy transfer (FRET) efficiency, which is enhanced at high temperature due to the fact that the polymer chains are collapsed/contracted leading to a decrease in the distance between donor and acceptor moieties. A good linear relationship between the values of I_555_/I_520_ and pH, the concentration of Fe^3+^ ions, and the temperature, was found; so it is concluded that the copolymer can be used as a multifunctional ratiometric chemosensor.

Qiao et al. reported recently a water-soluble fluorescent probe for intracellular temperature and Ca^2+^ sensing that combines a thermoresponsive polymer, curcumin, and Fluo-4 AM (a cell permeable calcium ion fluorescent dye). The simultaneous determination of Ca^2+^ concentration and temperature variation during the oxidative phosphorylation (OXPHOS) process has become the focus for exploration of signaling pathways and neurodegenerative disease [78]. The PNIPAM-VPBA-C-Fluo-4AM fluorescent polymer was successfully applied for intracellular temperature and Ca^2+^ gradient monitoring in HeLa cells. The authors report that within 10 min after the OXPHOS process was induced by an inhibitor, the temperature increased 0.5–1.0 °C and the Ca^2+^ level decreased by about 5.7 μM. The results confirmed that the fluorescent polymer used enabled the investigation of the relationship between intracellular temperature and Ca^2+^ induced neurotransmitter release [78].

Shen et al. reported a PNIPAM based K^+^ fluorescent sensor. A poly(NIPAM-co-MAA) copolymer was functionalized with a fluorescence potassium sensor (KS) based on 2-triazacryptand [2,2,3]−1-(2-methoxyethoxy) benzene (TAC), considered the best K^+^ chelator due to its reported high selectivity for detecting K^+^ over other metal cations [77]. The copolymer exhibited a lower critical solution temperature (LCST) at 38 °C, while its size changed in the range from 35 °C to 42 °C. The LCST affected the copolymer fluorescence intensities and responses to K+. Furthermore, the copolymer exhibited high selectivity and sensitivity to K^+^ with a dynamic response range from 1 to 20 mM, demonstrating its suitability for extracellular K^+^ analysis. The sensor was tested to in situ monitor the K^+^ fluctuation in *E. coli* and *B. Subtilis 168* bacteria, where a cell species dependent K^+^ release was observed. The PNIPAM-based K^+^ sensor showed its suitability in screening of antimicrobial drugs/peptides [77].

### 5.5. Theranostics

The ability to simultaneously detect as well as treat diseases is very appealing and may expand the versatility of therapeutic platforms available for nanomedicine. The field of theranostics has attracted a lot of interest since it offers the possibility of delivering the drug while simultaneously monitoring its biodistribution and therapeutic response. Polymer-based nanomaterials are the most prominent building blocks for construction of multi-functional theranostic agents due to their excellent biocompatibility, degradability, and structural and compositional versatility. The combination of thermoresponsive and fluorescent polymers is a special combination that offers a great potential of application in theranostics. To date, few nanosystems of this kind have been developed and tested.

Chambre et al. reported the preparation of multi-functional nanogels as theranostic platforms. In their approach a thermoresponsive copolymer is first prepared, then its response temperature is used for self-assembly. The nanosized aggregates were subsequently cross-linked with dithiols using thiol-maleimide chemistry to yield nanogels containing thiol, maleimide, and hydroxyl groups [66]. The hydroxyl groups were further activated to carbonates for conjugation with amine-containing molecules through carbamate linkage under mild conditions. The authors demonstrated multi-functionalization of the nanogels, with a thiol-containing cancer cell targeting peptide, DOX as a drug, and a maleimide-containing fluorescent indocyanine Cy5 as a dye. The applicability of the prepared multifunctional nanogel for theranostics was tested against MDA-MB-231 breast cancer cells. The autofluorescence of DOX was used to follow cell internalization enhanced with the presence of the targeting peptide. Cell viability studies demonstrated that the multifunctionalized nanogel loaded with DOX was able to reduce the cell-viability to values below 50%, however it does so at a concentration much higher than free DOX.

In a different approach Deng et al. reported the preparation of a triblock copolymer consisting in a fluorescent block of PF, a thermoresponsive block of PNIPAM, and a hydrophilic block formed with OEGMA [14]. The resulting copolymer has the composition of PF_11_-*b*-PNIPAM_120_-*b*-POEGMA_17_, and because of its amphiphilicity, can self-assemble in an aqueous phase into a micelle consisting of a hydrophilic outer corona of POEGMA a thermosensitive middle shell of PNIPAM and a fluorescent inner core of PF. The self-assembled micelles were loaded with DOX and tested in HeLa cells (cervical cancer cells). Cell internalization was demonstrated, since the PF’s blue fluorescence is clearly observed after an incubation of 4 h; furthermore, a yellow color observed in a merged picture of stained cells suggests an efficient cellular uptake of the DOX-loaded micelles. Cell viability studies were used to determine the half-maximal inhibitory concentrations (IC_50_) of the DOX loaded micelles, resulting in much larger values as compared to those of free DOX, which may result from being DOX not effectively released from the micelles [14].

Lu et al. reported an interesting nanosystem constructed using conjugated polymers-based nanoparticles for combined chemo- and photodynamic (PDT) therapy using temperature-driven drug release, and fluorescence tracking of the nanosystem [81]. The nanoparticles were prepared by a self-assembly of temperature-responsive polymer PNIPAM, cationic conjugated poly(fluorene-co-vinylene) (PFV), and DOX. In the nano reprecipitation process, conjugated polymer PFV and DOX form the core by hydrophobic and π−π interactions, and a shell of the nanoparticles is formed by PNIPAM on the surface. The nanoparticles showed strong fluorescence, which provides the capability for cell imaging. The novelty of this approach is that PFV units not only help to track the system in cells by fluorescence, PFV acts as a photosensitizer to produce high reactive oxygen species under white light irradiation, bringing an effective PDT synergistic effect. The cell viability of MCF-7 breast cancer cells decreases to only 3.2% after treating with the conjugated polymer nanoparticles under white light irradiation for 72 h, which is much lower than that with a single treatment and also much lower than the effect of the free DOX under similar treatment conditions [81].

More recently, N. Pandey et al. reported the preparation and use of a thermoresponsive fluorescent polymer (TFP) conjugated on the surface of iron oxide MNPs for potential use in imaging and therapeutic applications on solid tumors [87]. The TFP was synthesized by copolymerizing PNIPAM, allylamine and a biodegradable photoluminescent polymer, prepared separately using polyethylene glycol and amino acids, showing a fluorescence emission at 532 nm. This TFP was then conjugated to silane functionalized MNPs via a FRP reaction. The TFP shell was thermoresponsive, fluorescent, degradable, and was able to load DOX and release it by temperature increase. The authors reported that the DOX-TFP-MNPs system was taken up by prostate and skin cancer cells and exhibited decreased viability of the cells at 41 °C. Preliminary in vivo studies showed theranostic capabilities of the nanoparticles with bright fluorescence, and therapeutic efficacy after systemic administration in tumor bearing mice. In vivo therapeutic efficacy of DOX-TFP-MNPs was studied in animals implanted with B16F10 skin tumors in the presence of a 1.3 T magnetic field (heating). At the end of the 15-day study, only an 11-fold increase in the original tumor volume was observed in animals treated with the DOX-TFP-MNPs in the presence of the magnetic field; while the control animals, given saline injections, showed a 61-fold increase in their original tumor volumes. The animals treated with empty TFP-MNPs and treated with free DOX showed 59-fold and 21-fold increases in their tumor volumes, respectively [87].

**Figure 9:**
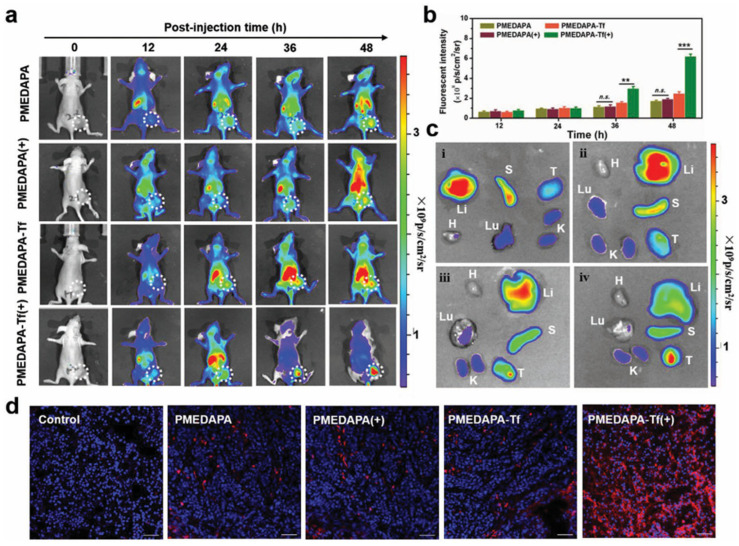
Fluorescent imaging of mice bearing HepG2 liver tumors. (**a**) In vivo fluorescence imaging of mice after intravenous injection (tumor pointed out with white circle). Postinjection of 36 and 48 h, microwave heating (2450 MHz per 8 W) is applied for 20 min before imaging, indicating PMEDAPA(+) and PMEDAPA-Tf(+). (**b**) Quantification of fluorescence intensities of tumor regions as a function of postinjection time after systemic administration. (**c**) Ex vivo imaging of tumor tissues and major organs at 48 h postinjection. (i) PMEDAPA, (ii) PMEDAPA(+), (iii) PMEDAPA-Tf, and (iv) PMEDAPA-Tf(+). T: tumor, H: heart, Li: liver, S: spleen, Lu: lung, K: kidney. (**d**) Confocal fluorescence images of tumor slices from mice after systemic administration at postinjection time of 48 h. Red fluorescence indicates nanogels and cell nuclei (blue) are stained by DAPI. Scale bar indicates 50 µm. n.s.: no significant difference. ** *p* < 0.01 and *** *p* < 0.001. Reprinted from reference [15], Copyright (2020), reproduced with permission from Wiley.

An impressive report on theranostic application of thermoresponsive-fluorescent nanomaterial was reported by S. Peng et al. A sulfamide-based zwitterionic monomer was prepared and used to synthesize a series of polysulfamide-based (poly (2-((2-(methacryloy-loxy)ethyl) dimethylammonio)acetyl) (phenylsulfonyl) amide (PMEDAPA) UCST based thermoresponsive nanogels as drug carriers for effective cancer therapy [15]. PMEDAPA nanogels were obtained after precipitation polymerization using ethylene glycol dimethacrylate (EGDMA) crosslinker and the addition of bis(acryloyl)cistamine (BAC) in small amount for further functionalization. PMEDAPA nanogels respond to hyperthermia by adjusting the crosslinker content. After being modified with transferrin (Tf), the nanogels (PMEDAPA-Tf) achieve tumor targeting at hyperthermia, leading to enhanced tumor accumulation. DOX-loaded PMEDAPA-Tf nanogels show superior penetration ability in 3D tumor spheroids and faster drug release at hyperthermia compared with that at normal temperature. This was revealed by fluorescence microscopy when the nanogels were further functionalized using a Cy5 tag. The DOX-loaded PMEDAPA-Tf nanogels show a pronounced tumor inhibition effect in a humanized orthotropic liver cancer model, as shown in Figure 9, when combined with mild microwave heating (20 min to achieve ≈41 °C) [15].

**Figure 10:**
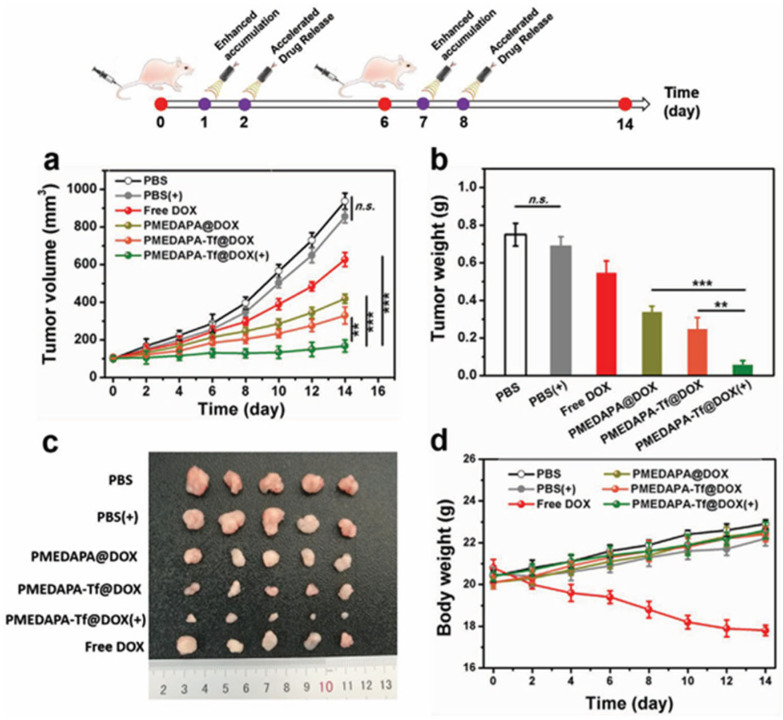
(**a**) Tumor growth curves, (**b**) weights, and (**c**) photograph of tumors after excision from each group with various treatments. (**d**) Weight change curves of HepG2 tumor-bearing mice post various treatments. Scale bar indicates 50 µm. +: microwave heating (2450 MHz per 8 W) for 20 min. n.s.: no significant difference. ** *p* < 0.01, and *** *p* < 0.001. Reprinted from reference [15], Copyright (2020), reproduced with permission from Wiley.

Fluorescent imaging of mice bearing HepG2 tumors showed that the functionalized nanogels were accumulated preferably in the tumor after microwave heating (20 min heating before imaging), although some accumulation in liver and spleen could not be avoided. Most important, the tumor was drastically reduced after 15 days of treatment (two injections, two heating cycles), a much better outcome than using free DOX or any another combination without heating (Figure 10).

A very different approach was reported by Li et al. [88]. Elastin based polypeptides were used as thermoresponsive units for an inside cell transglutaminase enzyme catalyzed polymerization induced self-assembly. The authors report that the linearly grown elastin-like polypeptides were biocompatible and aggregate into spherical nanoparticles that exhibit significant molecular accumulation and retention effects, while 3D gel-like structures with thermo-induced multi-directional traction interfere with cellular fates, resulting in cell death. The theranostic potential of these nanoconstructs were highlighted by dying of the elastin-like polypeptides with a fluorescein isothiocyanate-peptide (FITC-peptide) for cell imaging and with cyanine-5 (Cy5)-labeled peptide for in vivo (HeLa tumor bearing mice) studies.

## 6. Conclusions and Perspectives

Thermoresponsive polymers containing fluorescent units have been prepared more frequently in the last ten years. Besides encapsulating or grafting onto fluorescent nanoparticles, the end-functionalization of polymeric nanoassemblies with organic dyes has been reported. Nevertheless, the most promising approach is the development of thermoresponsive block copolymers in which one block has organic fluorescent dyes incorporated. The fact that the thermoresponsive block in water can be changed from being hydrophilic to hydrophobic depending on temperature opens the possibility of self-assembly of the block copolymers to nanoconstructs like polymeric micelles, vesicles and, if a crosslinker is added in the synthetic scheme, to the formation of nano/microgels. This self-assembly phenomenon has been recently utilized for the study of very interesting fluorescent behavior like aggregation induced emission or the opposite, aggregation-induced quenching of fluorescence. The AIE behavior in particular has great potential for biological studies. Giving the vast number of organic dyes already available to attach to either block copolymers or monomers used to prepare block copolymers, polymeric nanoconstructs can be prepared showing fluorescence at different wavelengths, opening the door for a wide range of applications.

Up to now, the most studied applications are their use in biosensors, like nanothermometers, sensors for specific ions relevant for biological processes like Ca^2+^, K^+^, or even H^+^; and for tracking drug delivery systems in cells and organs. Specifically, in theranostic applications, there is a lot of interest of using smart polymeric materials. The combination of a thermoresponsive polymer with a fluorescent entity that is chemically attached to it, is very appealing if the system is also able to load and release drugs in a specific manner. However, only few reports to date have appeared in the literature.

Some drawbacks/opportunity areas from the already published reports in this regard, in our modest opinion, are:

1. The thermoresponsive polymer used in the majority of cases is PNIPAM, which is easy to polymerize and copolymerize well, but there is still some debate on its potential toxicity (to certain cells, aquatic life, among others) [89,90,91,92]. The use of other monomers that yield biocompatible thermoresponsive polymers like NVCL, OEGMA, and proteins are expected to grow in this kind of applications.

2. The self-assembly of block copolymers into micelles is well established; however, the preparation of vesicles, also called polymersomes, using thermoresponsive and fluorescent polymers is only scarcely reported and has great potential in cancer therapy, since many formulations in the market using anticancer drug delivery systems make use of liposomes.

3. For the tracking of drug delivery systems into cells, the use of organic dyes that emit in the visible range are a great choice and have been studied consistently; however, for the tracking of DDS in in vivo applications, the fluorescence emission in the NIR-region is needed. There are not many organic fluorophores that emit with enough intensity in the NIR-region (cyanines). In the literature, there are interesting approaches for this, like the use of gold nanorods, carbon dots, and the combination of organic molecules used as monomers and drugs that in complex emit in the NIR region. This area needs a lot of research.

4. The use of thermoresponsive polymers needs a heat trigger to yield either a drug release, an enhanced fluorescence emission and other effects. For this to be effective, physiological heating (fever) is not the best choice for on demand delivery; the combination of thermoresponsive and fluorescent polymers with gold nanoparticles (photothermal therapy), specific photosensitizers (photodynamic therapy), or magnetic nanoparticles (magnetothermal therapy) is in the research arena. These complex systems need to be tested and focused on in in vivo experiments to reveal their great potential for theranostic applications.

## Figures and Tables

**Table 1:** Summary of sort studies of thermoresponsive and fluorescent nanogels/microgels reported in the last 10 years.

Thermoresponsive Polymer	Fluorescent Unit	Emission (nm)	Polymerization Technique	Size (nm)	VPTT (°C) ^a^	Application	Ref.
PNIPAM	TVPA luminogen	500–700	PP	110 ^a,f^	32	Nanothermometer	[1]
PNIPAM	DBD-AA monomer	570–589	PP with cationic initiator	157 ^a,c^	30–35	Nanothermometer	[2]
PNIPAM	Benzothiadiazole	580	PP Cationic polymerization	22.3 ^d^	32	Nanothermometer	[3]
PNIPAM	Naphtalimide derivative	450	EP	304 ^a,c^	34–43	Sensors	[12]
PNIPAM	(1) Fluorescein (2) Rhodamine	(1)517 (2) 586	EP	173 ^a,g^	35.5	Sensors	[13]
PNIPAM	Pyranine	416–432	PP	140–550 ^b^	32	-	[65]
PNVCL-PEGDA PGMA-PCB	Rhodamine B	586	RAFT/SFEP	240–320 ^a,c^	33–37	Bioimaging	[9]
PDFEA/PHPMA or PMeOx	Fluorine atoms	600	RAFT	120 ^d^	30–50 ^e^	Bioimaging	[70]
PDEAEM-PEGMA	Fluorescein	520	SFEP	183–200 ^a,c^	40–45	Drug delivery	[6]
PNIPAM	Naphtalimide derivative	550	DP	10–30 ^a,c^	37	Drug Delivery	[17]
PNIPAM-PDMAEMA	Fluorescein	517	EP	98 ^a,c^	40	Drug delivery	[7]
PNVCL-PHPMA	(1) Fluorescein (2) Cyanine 7.5	(1) 517 (2) 800	PP	89–196 ^a,c^	35–44	Drug delivery	[8]
PMEDAPA	Cyanine 5	667	PP	130–200 ^a,c^	32–43	Theranostic	[15]
PEGMA/PFuMaMA/PHEMA	(1)BODIPY (2) Cyanine 5	(1) 605 (2) 715	RAFT	92 ^a,c^	60–70 ^h^	Theranostic	[66]

^a^ Determined by DLS; ^b^ Determined by AFM; ^c^ Measured at 25 °C; ^d^ Determined by TEM; ^e^ For polymers before nanogel synthesis; ^f^ Measured at 55 °C; ^g^ Measured at 40 °C; ^h^ Approximate value, used for nanogel synthesis.

**Table 2:** Summary of fluorescent temperature responsive copolymers and applications.

Copolymer	Synthesis Technique	Morphology	T_cp_ (°C)	Emission Region (nm)	Biological Assay	Application	Ref.
P(NIPAM-*co*-BODIPY-AA)	RAFT	Micelles	35	605	BHK cells	Nanothermometer	[4]
PEGMA-*b*-P(PNIPAM-*co*-NBDAE)	RAFT	Micelles	35	536	HepG2 cells	Nanothermometer	[5]
(PNIPAM)x-TPPEBr X = 1,2,3,4	FRP	Micelles	31–38	560	A549 cells	Nanothermometer	[75]
POx-Pyrene; POx-BODIPY; POx-Porphyrin	Cationic polymerization/Click react.	------	47, 43, 39	430, 520, 655	------	Sensors	[40]
P(NIPAM-*co*-BMPN-*co*-R6GEM)	RAFT	------	25–45	520 and 555	------	Sensors	[76]
P(NIPAM-*co*-MAA)-KS	FRP	Aggregates	38	572	------	Sensors	[77]
PNIPAM-VPBA-C-Fluo-4AM	RAFT	Micelles	35	738	HeLa cells	Sensors	[78]
P(NIPAM-*co*-TPE)-*b*-PDPA	RAFT	Micelles	36	470	MCF-7 cells	Bioimaging	[10]
PNIPAM-TPE-PNIPAM	ATRP	Micelles	34	480	HeLa cells	Bioimaging	[11]
P(NIPAM-*co*-TCMA) P(NIPMAM-*co*-BOBPYBX)	FRP	Aggregates	25–50	436 and 628	HeLa cells	Bioimaging	[48]
P(NIPAM-*co*-FL)	FRP	Aggregates	30	514	RAW264.7 cells	Bioimaging	[79]
Tyr-P(NIPAM-*co*-DMAA)-FL	RAFT	Aggregates	34–46	517	HeLa cells	Bioimaging	[80]
PEGMA-*b*-P(NIPAM-*r*-R6GMED)	RAFT	Vesicles	25	584	------	Drug delivery	[16]
PF-*b*-PNIPAM-*b*-POEGMA	Sequential click coupling-ATRP	Micelles	33	450	HeLa cells	Theranostic	[14]
PNIPAM-PFV	Heck coupling reaction	Aggregates	30–35	502	MCF-7	Theranostic	[81]
PMalpGP-*b*-POEGMA-*b*-P(Llys-*co*-Asp)	RAFT-ROP-Click reaction	Micelles	------	597 and 795	HepG2 and NIH3T3	Theranostic	[82]

## Data Availability

No applicable.

## Abbreviations

The following abbreviations are used in this manuscript:

A431 cells	Human epidermoid carcinoma cell line
A549 cells	Human lung adenocarcinoma cell line
ACQ	Aggregation-caused quenching
AFM	Atomic Force Microscopy
AIE	Aggregation-induced emission
AIEgens	AIE luminogens
AMMA	9-Anthrylmethyl methacrylate
ATRP	Atom transfer radical polymerization
B16F10 cells	Mouse melanoma cell line
BAC	*N,N*′ -bis(acryloyl)cystamine
BCPs	Block copolymers
BHK cells	Fibroblast-like cell line
BMPN	*N*-(n-butyl)-4-(4-[2-methacryloyloxyethyl]-piperazine-1-yl)-1,8-naphthalimide
BOBPYBX	Acrylated-dibenzoporphyrin derivative of BODIPY
BODIPY	4,4-difluoro-4-bora-3a,4a-diaza-s-indacene
B-R	Britton–Robinson
CMC	Critical Micellar Concentration
Cou	Coumarin
Cou-MMA	7-(2-methacryloyloxyethoxy)-4-methylcoumarin
CPCs	Colloidal photonic crystals
CPNs	Conjugated polymer nanoparticles
Cy	Cyanine
DA	Dansyl
DBD-AA	N-{2-(7-*N,N*-dimethylaminosulfonyl-2,1,3-benzoxadiazol-4-yl)methylamino}ethyl-*N*-methylacrylamide
D_h_	Hydrodynamic diameter
DLS	Dynamic Light Scattering
DMAA	*N,N*-dimethylacrylamide
DOPE	Dioleoyl
DOX	Doxorubicin
DP	Dilution polymerization
DPA	2-(Diisopropyl amino)ethyl methacrylate
EE	Encapsulation efficiency
EP	Emulsion polymerization
EPR	Enhanced permeation and retention
FIM	*N*-2-(6-(4-methylpiperazin-1-yl)-1,3-dioxo-1H-benzo[de]isoquinolin-2(3H)-yl-ethyl)acrylamide
FITC	Fluorescein isothiocyanate
FL	poly[*N*-(2,2difluoroethyl)acrylamide]
Flu	fluorescein
Flu-DA	Fluorescein-*O,O*’-diacrylate
Flu-MMA	Fluorescein methylmethacrylate
Flu-NGs	Fluorescein-labeled PNVCL-PHPMA nanogels
Fluo-4 AM	Cell permeable calcium ion fluorescent dye
FRET	Förster-type resonance energy transfer
FRP	Free radical polymerization
G361 cells	Human melanoma cell line
GSH	Glutathione
GTP	Group Transfer Polymerization
Hb	Hemoglobin
HbO2	Hemoglobin oxidized
HeLa cells	Cervical cancer cells
HepG2 cells	Human liver cancer cell line
HPMA	*N*-(2-hydroxypropyl)methacrylamide
IC50	half-maximal inhibitory concentration
KS	Potassium fluorescent sensor
LAT1	L-type amino acid transporter 1
LCST	Lower critical solution temperature
MAA	Methacrylic Acid
MAlpGP	6-O-Methacryloyl-1,2:3,4-di- O-isopropylidene-D-galactopyranose
MCF-7	Human breast adenocarcinoma
MNPs	Magnetic nanoparticles
MTS	3-(4,5-dimethylthiazol-2-yl)-5-(3-carboxymethoxyphenyl)-2-(4-sulfophenyl)-2H-tetrazolium
MTT	3-(4,5-dimethylthiazol-2-yl)-2,5-diphenyltetrazolium bromide
Naphtalimide-AA	Acrylamide derivative of naphtalimide
NBDAE	2-Acryloyloxyethylamino-7-nitro-2,1,3-benzoxadiazole
NIR	Near-infrared
NIR-II	Second near-infrared
NMRP	Nitroxide-Mediated Radical Polymerization
NVCz	9-Vinylcarbazole
OEGMA	Oligoethyleneglycol methacrylate
OXPHOS	Oxidative phosphorylation
PC3 cells	Human prostate cancer cell line
PCB	Poly(carboxybetaine)
PDPA	Poly(diisopropyl amino) ethyl methacrylate
PDEAEM	Poly(*N,N*-diethylaminoethyl methacrylate)
PDFEA	Poly[*N*-(2,2difluoroethyl)acrylamide]
PDI	Polydispersity index
PDMAEMA	Poly(*N,N*-dimethylamynoethyl methacrylate)
PEGDA	Poly(ethyleneglycol diacrylate)
PEGMA	Poly(ethylene glycol) monomethyl ether methacrylate
PET	Photoinduced electron transfer
PF	Polyfluorene
PFV	Poly(fluorene-co-vinylene)
PFuMaMA	Furan-protected maleimide-containing methacrylate
PGMA	Poly(glycidyl methacrylate)
PHEMA	Poly(2-hydroxyethyl methacrylate)
PHPMA	Poly[*N*-(2-hydroxypropyl)-methacrylamide]
PL	Photoluminescence
P(Llys-co-Asp)	Poly(L-lysine-co-aspartate)
PMEDAPA	Poly(2-((2-(methacryloyloxy)ethyl) dimethylammonio) acetyl) (phenylsulfonyl) amide
PMeOx	Poly(2-methyl-2-oxazoline)
PNIPAM	Poly(*N*-isopropylacrylamide)
PNIPMAM	Poly(*N*-isopropylmethacrylamide)
PNVCL	Poly(*N*-vinylcaprolactam)
POEGMA	Poly(oligo(ethylene glycol) monomethyl ether methacrylate)
POx	Poly(2-isopropyl-2-oxazoline)
PP	Precipitation polymerization
PyMA	1-Pyrenemethyl methacrylate
R6GMED	*N*-(rhodamine-6G)lactam-*N*′-methacryloyl ethylenediamine
RAFT	Reversible addition–fragmentation chain transfer
RH	Rhodamine
RIM	Restriction of intramolecular motion
RIR	Restriction of intramolecular rotation
RIV	Restriction of intramolecular vibration
SFEP	Surfactant free emulsion polymerization
TAC	2-triazacryptand [2,2,3]-1-(2-methoxyethoxy) benzene
TCMA	7-[4-(Trifluoromethyl)coumarin]methacrylamide
T_cp_	Cloud Point Temperature
TEM	Transmission Electron Microscopy
Tf	Transferrin
TFP	Thermoresponsive fluorescent polymer
TPE	Tetraphenylethene
tPE	Triphenylethene
TPE-MMA	Tetraphenylethene methyl methacrylate
TPPEBr	Tetraphenylene fluorophore
TVPA	Acrylated AIEgen with electron-donating triphenylamine and electron-withdrawing pyridinium unit connected by an ethylene core.
UCST	Upper critical solution temperature
VF	9-Vinyl-9H-fluorene
VPBA	4-Vinylphenyl boronic acid
VPTT	Volume phase transition temperature
